# Novel insecticide-removal activity against deltamethrin and malathion in a lithic-derived *Modestobacter* strain

**DOI:** 10.3389/fmicb.2026.1871101

**Published:** 2026-07-29

**Authors:** Tasnim Hajri, Ramona Marasco, Ilhem Saadoouli, Khouloud Hammami, Wafa Babay, Hanen Cherif, Soumaya Kouidhi, Hadda-Imene Ouzari, Meriem Msaad, George Tsiamis, Ameur Cherif, Amor Mosbah

**Affiliations:** 1 Laboratory of Biotechnology and Bio-Geo Resources Valorisation (LR11ES31), Higher Institute of Biotechnology of Sidi Thabet (ISBST), University of Manouba, Tunis, Tunisia; 2 Biological and Environmental Sciences and Engineering Division, King Abdullah University of Science and Technology, Thuwal, Saudi Arabia; 3 Laboratory of Microorganisms and Active Biomolecules (LR03ES03), Faculty of Sciences of Tunis (FST), University Tunis El Manar, Tunis, Tunisia; 4 Laboratory of Biotechnology and Nuclear Technologies (LR16CNSTN01), National Centre for Nuclear Science and Technology, Tunis, Tunisia; 5 Laboratory of Systems Microbiology and Applied Genomics, Department of Sustainable Agriculture, University of Patras, Agrinio, Greece

**Keywords:** actinobacteria, bioremediation, byproduct toxicity, deltamethrin, malathion, nature-based solution, pesticide degradation

## Abstract

A large fraction of agricultural pesticides, including insecticides, fails to reach target organisms and instead persists in soil, where residues may accumulate and generate transformation products with unpredictable or enhanced toxicity. Therefore, sustainable mitigation strategies require not only efficient pesticide removal but also effective detoxification. Soil-dwelling bacteria, particularly actinobacteria, possess diverse enzymatic capabilities that can contribute to the transformation of these chemical compounds, thereby influencing their environmental fate. In this study, we evaluated the insecticide-removal potential of 70 actinobacterial strains isolated from oligotrophic environments (dust and stone surfaces) in Tunisia using deltamethrin (a pyrethroid) and malathion (an organophosphate) as model insecticides. Thirty-two strains grew in minimal medium containing 3.2 mg/L of at least one insecticide, with 14 strains growing on both compounds. Among them, the actinobacterium *Modestobacter marinus* strain G96 (order *Geodermatophilales*) exhibited the highest apparent insecticide removal, as quantified by high-performance liquid chromatography. The concomitant increase in bacterial growth, together with the absence of growth in insecticide-free minimal medium, indicates that the apparent removal of parent insecticides is attributable to bacterial proliferation rather than to passive adsorption alone. Incubation of insecticide with G96 also resulted in reduced toxicity, with malathion-treated supernatants becoming completely non-toxic to *Artemia salina* by day 14. Malathion apparent removal was further assessed in a complex edaphic substrate (i.e., soil-slurry microcosms), where the concentration of the parent compound decreased by 90% over the 21-day incubation. Overall, our findings identify *M. marinus* G96 as a promising candidate for the biological removal and detoxification of insecticides and highlight the biotechnological potential of extremotolerant actinobacteria for the sustainable remediation of pesticide-contaminated soils, particularly in arid and dryland ecosystems.

## Graphical Abstract

**Figure:**
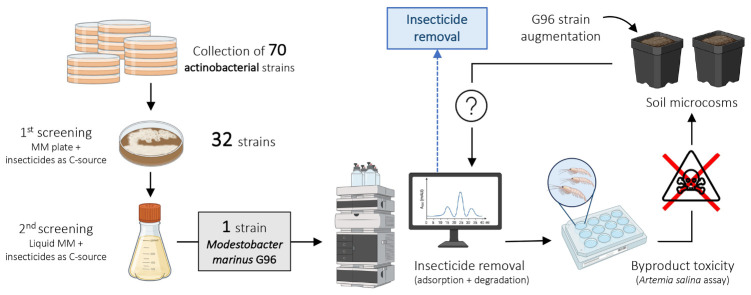
Screening and selection of lithic actinobacterial strains for insecticide removal. A collection of 70 actinobacterial strains was screened on solid and liquid minimal media supplemented with insecticides as the sole carbon source. *Modestobacter marinus* strain G96 was selected based on its insecticide removal performance. Further assays evaluated its ability to reduce the toxicity of degradation by-products using the *Artemia salina* bioassay and to remove malathion in soil microcosms, demonstrating its potential for bioremediation applications.

## Introduction

Synthetic chemical pesticides, including insecticides, remain the most extensively used agents for agricultural pest control (Bondareva and Fedorova, 2021), with a global consumption approaching 3 million tons per year (FAO, 2023). They are grouped into major chemical classes, including organochlorines, organophosphates, carbamates, pyrethroids, phenylamides, triazines, and benzoic acid derivatives, each characterized by distinct molecular structures and mechanisms of action (Rani et al., 2020).

While the use of pesticides has undeniably contributed to increased food production and effective pest control, it has also resulted in unintended, far-reaching environmental consequences (Samada and Tambunan, 2020). Less than 5% of applied pesticides reach their intended target organisms (Kumar et al., 2019; Vasseghian et al., 2022), while the remaining disperse into soils, water bodies, and the atmosphere, where they persist as residues or undergo partial (or in some cases total) biological degradation (Maggi et al., 2023). Recent meta-analyses encompassing more than 1,700 studies have shown that all major pesticide classes, i.e., herbicides, insecticides, and fungicides, exert toxic effects on a wide range of non-target organisms, including beneficial microorganisms, plants, invertebrates, fish, birds, and mammals (Serrão et al., 2022; Wan et al., 2025). These effects have severe implications for ecosystem integrity (Yadav and Devi, 2017; Tudi et al., 2021; Zhou et al., 2025) and food safety (Lazarević-Pašti et al., 2025). Besides these efforts, the decontamination of pesticide-polluted environments remains a crucial aspect and challenge that needs to be addressed. Conventional remediation strategies, including chemical neutralization, incineration, and physical removal, are often costly, energy-intensive, and may generate secondary pollutants (Chaturvedi and Khurana, 2019). More recently, advanced physicochemical technologies, such as electrocatalytic oxidation, have demonstrated high removal efficiencies for organophosphorus compounds and a range of other organic contaminants, including antibiotics (Li et al., 2025; Wang et al., 2026). However, these approaches generally require specialized infrastructure, external energy inputs, or chemical reagents, which may limit their application for large-scale or *in situ* remediation. Biological remediation instead offers a complementary and environmentally sustainable alternative by exploiting the metabolic capabilities of microorganisms and their enzymes to transform xenobiotic compounds through biodegradation, producing less toxic intermediates or, in some cases, complete mineralization (Huang and Lu, 2021). Unlike physicochemical approaches, microbial biodegradation can be self-sustaining under environmentally relevant conditions and may simultaneously contribute to contaminant removal and detoxification.

Numerous edaphic and aquatic microorganisms, including species of *Pseudomonas* (Gilani et al., 2016), *Bacillus* (Arora, 2020), and members of the phylum *Actinomycota* (Schrijver and Mot, 1999), have been shown to metabolize a wide range of pesticides, thereby reducing their persistence and toxicity in the environment (Jose et al., 2021; Ravi and Gupta, 2023). Advances in metagenomics have further enabled the identification and enhancement of key degradative pathways, including hydrolases, oxygenases, and dehalogenases, that target specific functional groups in pesticide molecules (Mishra et al., 2021). Among the most promising microbial degraders are actinobacteria, which are both widely distributed in soil and water ecosystems and are recognized for their broad-spectrum catabolic potential, including the ability to mineralize persistent xenobiotics (Zhang et al., 2020; Ravi and Gupta, 2023). Beyond enzymatic versatility, actinobacteria possess ecological adaptations that further enhance their bioremediation potential (Alvarez et al., 2017), including tolerance to nutrient limitation, desiccation and chemical stress, together with production of biosurfactants that can enhance the bioavailability of hydrophobic contaminants (Shivlata and Satyanarayana, 2017; Boubekri et al., 2022).

Despite this recognized potential (Alvarez et al., 2017; Shoba et al., 2022; Rebai et al., 2024), isolating and selecting efficient actinobacterial strains remains a major bottleneck, as it typically requires extensive culture-dependent screening and validation. In this work, we reasoned that targeting a nutrient-poor ecological niche could help offset this screening burden, as actinobacteria inhabiting such environments are expected to possess broad metabolic plasticity and enzymatic repertoires adapted to exploiting chemically diverse and often recalcitrant carbon sources under chronic nutrient limitation (Pointing and Belnap, 2012; Marasco et al., 2022). Such metabolic flexibility may also confer the capacity to utilize and/or transform structurally unrelated anthropogenic xenobiotics, including pesticides. For this purpose, we screened a collection of 70 actinobacterial isolates recovered from lithic substrates and dust across Tunisian drylands (Saadouli et al., 2022) for their ability to grow in minimal medium supplemented with two insecticides separately, i.e., deltamethrin and malathion, both of which are widely used in agriculture and public health. When consistent, progressive bacterial growth was observed, we subsequently evaluated the apparent removal of the parent insecticide in liquid culture and soil-slurry microcosms, together with the associated changes in toxicity using *Artemia salina* as a model organism. By identifying extremotolerant actinobacteria capable of reducing insecticide loads under nutrient-limited conditions, our work provides new insights into their metabolic potential that may contribute to the development of sustainable bioremediation strategies for pesticide-contaminated soils and water.

## Materials and methods

### Insecticide selection

Deltamethrin and malathion were selected due to their extensive use in agriculture and public health, as well as for their contrasting chemical classes and modes of action (Toumi et al., 2018). Deltamethrin([(S)-α-cyano-3-phenoxybenzyl] (1R,3R)-3-(2,2-dibromovinyl)-2,2-dimethylcyclopropane-1-carboxylate; CAS number 52918-63-5; Supplementary Figure 1A) is a widely used type II synthetic pyrethroid pesticide applied in agricultural, medical, and household settings (Lu Q. et al., 2019; Pitzer et al., 2021; Shi et al., 2024). It is non-volatile, lipophilic, and chemically stable, with a low vapor pressure of 2.0 × 10^–6^ Pa (1.5 × 10^–8^ mm Hg). While it is highly soluble in organic solvents such as kerosene, acetone, and xylene, it is nearly insoluble in water (<0.2 mg/L) (Rehman et al., 2014).

Instead, malathion (diethyl 2-[(dimethoxy phosphorothioyl)sulfanyl]butanedioate; CAS number 121-75-5; Supplementary Figure 1B) is a quasi-systemic organophosphate insecticide widely used to control agricultural pests, such as aphids and grasshoppers, and vector mosquitoes responsible for disease transmission (Kaur et al., 1997; Pupim et al., 2023). It acts primarily by inhibiting acetylcholinesterase (Bray et al., 2021) and is relatively water-soluble, with hydrolysis rates increasing at higher pH (Jensen and Whatling, 2010).

Both compounds are known to persist in soils, where they can accumulate and affect non-target organisms (Song et al., 2015; Dar and Kaushik, 2023), making them suitable candidates for evaluating the potential for bacterial biodegradation.

Deltamethrin (99.5% pure) and malathion (96% pure) were obtained from the National Center for Nuclear Science and Technology (Tunis, Tunisia).

### Screening of actinobacteria capable of growing on insecticides as sole carbon sources

The 70 actinobacterial strains tested in this study are part of a bacterial collection obtained from rock samples and dust collected from various regions of Tunisia. Details of the isolation, maintenance, and identification procedures, as well as the corresponding GenBank accession numbers (ON13902–ON713972), are reported in Saadouli et al. (2022). The bacterial strains were routinely cultured in Luedemann medium containing 5 g/L NaCl, 10 g/L glucose, 10 g/L malt extract, 5 g/L yeast extract, 5 g/L starch, 2 g/L CaCO_3_, and 18 g/L agar. To assess their ability to grow on deltamethrin and malathion as the sole supplemented complex carbon source, bacteria were streaked on solid minimal medium (MM) containing 0.5 g/L L-asparagine, 0.5 g/L K_2_HPO_4_, 0.2 g/L MgSO_4_⋅7H_2_O, 0.01 g/L FeSO_4_⋅7H_2_O, and 15 g/L agar at a pH of 7.3 (Hopwood, 1967) and supplemented with 3.2 mg/L of one of the two insecticides (Fuentes et al., 2010) after the autoclave sterilization process. The insecticides were dissolved in ethanol (0.1%) and sterilized by filtration through a 0.22 μm filter before addition. Control plates consisting of MM without insecticide supplementation were also included as negative controls to determine whether any component of the medium or the agar could support bacterial growth. Inoculated plates were incubated at 30 °C for 7–10 days, and bacterial growth was assessed by colony development. Positive growth was recorded when a homogeneous colony patina covered the entire streak.

### Quantification of the apparent removal of parent insecticides in liquid culture

Actinobacterial strains showing positive growth on insecticide-supplemented MM agar plates were subsequently evaluated in liquid MM cultures to quantitatively assess bacterial growth and the apparent removal of the parent insecticides over time (i.e., the experimentally measured decrease in the parent insecticide concentration due to adsorption and/or biodegradation). The selected bacteria were first precultured in liquid Luedemann medium at 30 °C for 24–72 h with shaking at 120 rpm. Five mL of each preculture was inoculated into tubes containing 15 mL of MM broth supplemented with 3.2 mg/L of either deltamethrin or malathion. Cultures were incubated at 30 °C with shaking (120 rpm), and samples were collected hourly during the first 4 h, then every 24 h for up to 72 h. Bacterial growth was monitored by measuring the optical density at 600 nm (OD_600_), with non-inoculated MM medium supplemented with insecticide serving as a control. During the initial optimization of the experimental protocol, the suitability of OD_600_ as a proxy for bacterial growth was verified by plating 100 μL aliquots of representative bacterial cultures onto rich-medium agar plates at different incubation times. Colony formation confirmed that the progressive increase in OD_600_ reflected an increase in viable bacterial cells under the experimental conditions. Based on this, OD_600_ was used as the growth-monitoring parameter for all subsequent experiments.

To quantify insecticides, initial UV–Vis absorbance spectra were recorded over a broad wavelength range (200–800 nm) to identify the absorption maxima of the compound solutions, which were observed between 214 and 290 nm. Culture samples were then centrifuged at 4000 rpm for 10 min, and the resulting supernatants were filtered through 0.22-μm syringe filters into sterile tubes. High-performance liquid chromatography (HPLC) analysis was conducted under isocratic conditions for 15 min, using a methanol–water mobile phase (80:20, v/v) at a flow rate of 0.8 mL/min and a column temperature of 37 °C. For each sample, a 20 μL aliquot was injected into a Shimadzu UFLC system equipped with an Intersil ODS-4 C18 column (4.6 × 250 mm, 5 μm). Analytes were detected by UV–Vis absorbance within the wavelength range of 214–290 nm. Quantification of insecticides was performed by comparing the sample chromatographic peak areas with those obtained from calibration curves. These calibration curves were constructed using known concentrations of each insecticide, allowing the corresponding concentrations of the concentrations to be interpolated from their peak areas. The amount of insecticide removed/degraded was then determined by subtracting the measured concentration from the initial concentration (see HPLC analytical method validation). Finally, bacterial growth (OD_600_) and insecticide reduction were plotted against time (0–72 h), while their relationship was assessed using simple linear regression in GraphPad Prism 10.

### HPLC analytical method validation

To ensure accurate and reliable quantification of insecticide concentrations throughout this study, the analytical performance of the HPLC method was validated prior to sample analysis. The sensitivity of the chromatographic system was assessed by calculating Sandell’s sensitivity (SS; g/cm^2^ per 0.001 absorbance unit). In addition, the Limit of Detection (LOD) and the Limit of Quantification (LOQ) were determined to evaluate the method’s sensitivity for detecting and quantifying deltamethrin and malathion residues. These parameters were calculated from the standard deviation of the y-intercept (σ) and the slope (S) of the calibration curves according to the equation: LOD = 3.3 × (σ/S) and LOQ = 10 × (σ/S) (Abdelwahed et al., 2022). The complete validation results, including calibration parameters, Sandell’s sensitivity thresholds, LOD, and LOQ for both insecticides, are reported in Supplementary Table 1. Analytical recovery was assessed using abiotic controls consisting of culture medium containing the pesticides but no bacterial inoculum. Abiotic controls were processed identically to inoculated samples, including extraction, sample preparation, and HPLC analysis, to account for analyte losses throughout the analytical procedure. The percentage of recovery was calculated as [(measured concentration in the abiotic control)/(nominal concentration added)] × 100. Notably, the analytical recovery ranged from 98% to 99% for both pesticides.

### Growth and apparent removal capacities of strain G96 at increasing pesticide concentrations

Among the screened actinobacterial strains, *Modestobacter marinus* G96 emerged as the most efficient at removing the target insecticide. Given that such metabolic potential had not been reported previously for this genus, we further investigated its degradation dynamics, activity, and concentration thresholds. The same protocol described above for evaluating bacterial growth and insecticide degradation was applied, but with increasing initial insecticide concentrations (16 and 128 mg/L) added to the MM medium. Specifically, the G96 strain was cultivated in 100 mL flasks containing 50 mL of Ludemann medium and incubated at 30 °C with continuous shaking at 120 rpm for 3 days in the dark. After incubation, a 100 μL aliquot of the culture was suspended in 900 μL sterile physiological solution (0.9% NaCl), followed by a series of tenfold serial dilutions. A 20 μL sample from the final dilution was then transferred to a Malassez counting cell for measuring bacterial density: *N* = [*n*/(*a* × *v*)] × *Fd*, (*N*: number of cells per unit volume; *n*: number of cells counted; *a*: number of counting units counted; *v*: volume of a counting unit; *Fd*: dilution factor). Five mL containing approximately 10^6^ cells/mL was incubated with deltamethrin or malathion at concentrations of 3.2, 16, and 128 mg/L. Cultures were monitored over a 16-day incubation period at 30 °C. Bacterial growth (OD_600_) and the reduction of parent insecticides in the medium were measured over 16 days using the same methodology as in short-incubation experiments (*n* = 2 per condition). OD_600_ and insecticide concentration reduction were plotted against time (0–16 days), and their relationship was evaluated using simple linear regression in GraphPad Prism 10.

### Evaluation of changes in insecticide toxicity following incubation with G96

*Artemia salina* was used to evaluate the toxicity of byproducts resulting from the modification of the insecticides during the incubation with G96 as reported by Samri et al. (2015). A. salina cysts (around 0.5 g) purchased from JBL Artemio Pur were placed into a vial containing 500 mL of sterile seawater and incubated at 29 ± 1 °C, with aeration provided by an air pump and illumination by lamps placed near the hatching containers, maintaining a photoperiod of 16:8 (light/dark). After 72 h of incubation, the hatched larvae (nauplii) displaying phototactic behavior were collected and transferred into separate wells of a 48-well plastic tissue culture plate for toxicity testing, with 30 larvae per well. The wells were then treated with 100 μL of filtered supernatant from (i) bacterial culture of G96 strain grown in MM with deltamethrin (3.2 mg/L) or malathion (16 mg/L, i.e., the highest concentration at which degradation was observed for this strain; see Results), (ii) bacterial culture of G96 strain grown in Luedemann medium, and (iii) MM medium with insecticides without inoculation of G96 strain; all treatments were prepared in triplicates, i.e., 3 independent flasks each. Filtered solution from the cultures and medium at days 0 (less than 30 min after inoculation), 7, and 14 was used for the assay. Seawater containing the larvae served as the negative control. All experiments were performed in triplicate (*n* = 3 per treatment at each time point). At each sampling time, *A. salina* larvae were exposed to the three treatments for 24 h, after which mortality was assessed by recording individuals that showed no movement after gentle agitation as dead (Tzima et al., 2022); a short incubation period was used to assess acute toxicity, since prolonged exposure would introduce additional confounding factors unrelated to insecticide exposure, such as changes in larval nutritional status, developmental stage, and natural mortality, that could obscure the specific toxicological effect under investigation. The formula used for calculating the percentage of mortality was:


$$M\left(\%\right)=\frac{N}{T}\times 100$$

*M*: Mortality percentage; *N*: Number of dead *Artemia salina*; *T*: Total number of *A. salina*.

Each time point was evaluated independently using a new group of 30 larvae. Differences among treatments and incubation times were analyzed using a generalized linear model (GLM) with a binomial error distribution and logit link function. The response variable was specified as the number of dead and surviving larvae [cbind(dead, alive)], with treatment and incubation time included as explanatory variables. The significance of each factor was evaluated using likelihood-ratio chi-square tests. The interaction between treatment and incubation time was initially evaluated but was not retained in the final model because the presence of complete separation (i.e., certain treatment groups exhibiting either 0% or 100% mortality, see Section “Results”) prevented reliable estimation of the interaction coefficients in the binomial GLM. Consequently, the final model included only additive main effects (i.e., treatment and time) for the supernatant from bacterial cultures with insecticides. Pairwise comparisons of incubation times in the treatment of culture supernatant in the presence of bacteria and insecticide were performed using Fisher’s exact tests with Holm correction for multiple comparisons. Fisher’s exact tests were selected because some comparisons exhibited complete mortality (100%) or complete survival (0%), preventing reliable estimation of post hoc contrasts using GLM-based procedures. Statistical analyses were performed in R (version 2024.09.1), and differences were considered statistically significant at *P* < 0.05.

### Assessment of apparent parent malathion removal in soil-slurry microcosms

To provide an initial assessment of the removal potential of strain G96 in a complex soil matrix, experiments were conducted using microcosms containing a soil resuspension in a liquid medium. Soil slurry systems are widely employed in laboratory-scale screening and *ex situ* bioremediation studies, in which contaminated soils are excavated and treated under controlled conditions by mixing them with water or nutrient solution in continuously agitated reactors (Robles-González et al., 2008; Azubuike et al., 2016). These systems are specifically designed to maximize contact between microorganisms and contaminants by maintaining a homogeneous suspension of soil particles, thereby improving oxygen transfer, nutrient availability, and contaminant bioavailability (Marín-Benito et al., 2017). The soil-slurry microcosms were prepared using 50 g of soil and 50 mL of bacterial culture (n = 2 per treatment). Specifically, the soil was collected from Sidi Thabet (N 36° 54’ 31″, E 10° 02’ 33″, Altitude 324 m), Ariana, Tunisia, at a depth of 30 cm (Supplementary Table 2). In the laboratory, it was air-dried on glass plates, crushed, and sieved through a 2 mm sieve. Microbial load was reduced by autoclaving the soil three times at 120 °C for 20 min each. The *M. marinus* G96 strain was cultivated in 100 mL flasks containing 50 mL of Ludemann liquid medium at 30 °C with continuous shaking at 120 rpm for 3 days. Following cell counting with a Malassez counting cell, solutions containing 5 × 10^8^ or 1 × 10^9^ cells were prepared and added to the flasks containing soil, with malathion added at concentrations of 64 and 128 mg/kg. We focused on malathion because it was the insecticide most extensively removed by G96 when supplied as the sole carbon source (see Section “Results”). We deliberately spiked concentrations that exceeded the modeled worst-case soil concentration (to expose bacteria to potential localized contamination hotspots, such as pesticide spill sites, storage/dump areas, and formulation facilities (Silva et al., 2019). In addition, because soil is a structurally and chemically complex matrix containing indigenous organic matter and co-occurring carbon sources, bacterial metabolic activity can be supported independently of malathion, potentially extending the range of removal via co-metabolism beyond what is achievable under nutrient-limited, single-substrate conditions (Srivastava et al., 2026).

The soil-slurry microcosms with the different combinations of malathion and bacterial inocula were incubated in a shaker at 30°C and 120 rpm for 21 days. Two abiotic controls without bacterial inoculation were included: the first consisted of soil amended with malathion to determine the analytical recovery of the parent insecticide, whereas the second consisted only of soil slurry to verify that endogenous soil constituents did not interfere with the HPLC detection and quantification of the parent compound. Extraction of the residue parent insecticide was performed at 7, 14, and 21 days. For extraction, a methanol-chloroform mixture (50:50) was added to the soil-slurry samples, vortexed for 5 min, and then sonicated in an ultrasonic bath for 30 min. Subsequently, the samples were centrifuged at 5,500 rpm for 15 min, and the supernatants were collected into Falcon tubes. These tubes were processed using a rotary evaporator to completely remove the solvents, yielding a crude extract, which was then resuspended in 1 mL of methanol, filtered, and analyzed by HPLC as previously described. Differences in reduction rates over time within each bacterial concentration and insecticide treatment were analyzed using the nonparametric Kruskal–Wallis test or the Mann–Whitney U test, depending on the number of groups being tested. Statistical analyses were performed using GraphPad Prism 10.

### *In silico* prediction of the malathion degradation pathway in strain G96

To infer the putative degradation pathway of malathion by *M. marinus*, its genome was retrieved from the NCBI Datasets (2020) (GenBank assembly GCF_014645755.1). As the degradation pathway of malathion by the *Modestobacter* genus is not currently annotated in the KEGG database^1^, we potentially inferred the pathway annotated in *Arthrobacter* (family *Micrococcaceae*^2^) to identify possible enzymatic steps and intermediates. The selection of this strain was based not only on its classification within the actinobacteria but also on the presence of analogous enzymes encoded in the *M. marinus* genome (Supplementary Data S1).

We retrieved the sequences of all enzymes involved in malathion degradation from KEGG and performed a BLAST search for sequence similarity. The sequence with the highest similarity score was chosen for molecular modeling using I-TASSER (Yang and Zhang, 2015) to determine the three-dimensional (3D)structures of the candidate enzymes. Similarly, the 3D structures of the metabolites were sourced from PubChem. The resultant models were analyzed using AutoDock Tools and AutoDock Vina (Trott and Olson, 2009) to predict the optimal binding positions, conformations, and binding energies of each substrate interacting with its respective enzyme during malathion degradation. The most favorable solutions were visualized with PyMol (Yuan et al., 2016).

## Results

### Selection of actinobacteria growing on insecticides as the sole carbon source

After 10 days of incubation on MM agar plates supplemented with the test insecticides, 32 of the 70 actinobacterial isolates exhibited growth on at least one insecticide. Specifically, 9 strains grew exclusively on deltamethrin, 9 exclusively on malathion, and 14 on both insecticides (Supplementary Table 3). The remaining strains either exhibited negligible growth or were inhibited by the insecticides (representative examples are shown in Supplementary Figure 2A,B). To determine whether bacterial growth was associated with the presence of the insecticides rather than other components of the medium, the isolates were also cultured on MM without insecticide supplementation. Of the 32 isolates that grew in the presence of at least one insecticide, 18 strains showed no detectable growth on the insecticide-free control medium (Supplementary Figure 2C and Supplementary Table 3), suggesting that the insecticides were used as a primary carbon source for these isolates under the tested conditions.

### Quantification of apparent insecticide removal by actinobacteria

Among the actinobacteria that showed positive growth on one or both insecticides, 15 strains for which no prior evidence of malathion or deltamethrin transformation and/or degradation had been reported in the literature were selected to quantify their parent-insecticide removal capacity in liquid culture. Notably, the selected strains included isolates that showed no detectable growth on MM without insecticide supplementation (i.e., the strongest candidates for insecticide-dependent growth) and isolates that also grew on the insecticide-free control medium, which were intentionally retained to assess whether insecticide removal also occurs in strains capable of utilizing alternative carbon sources, consistent with co-metabolic degradation (Cycoń and Piotrowska-Seget, 2016).

**FIGURE 1:**
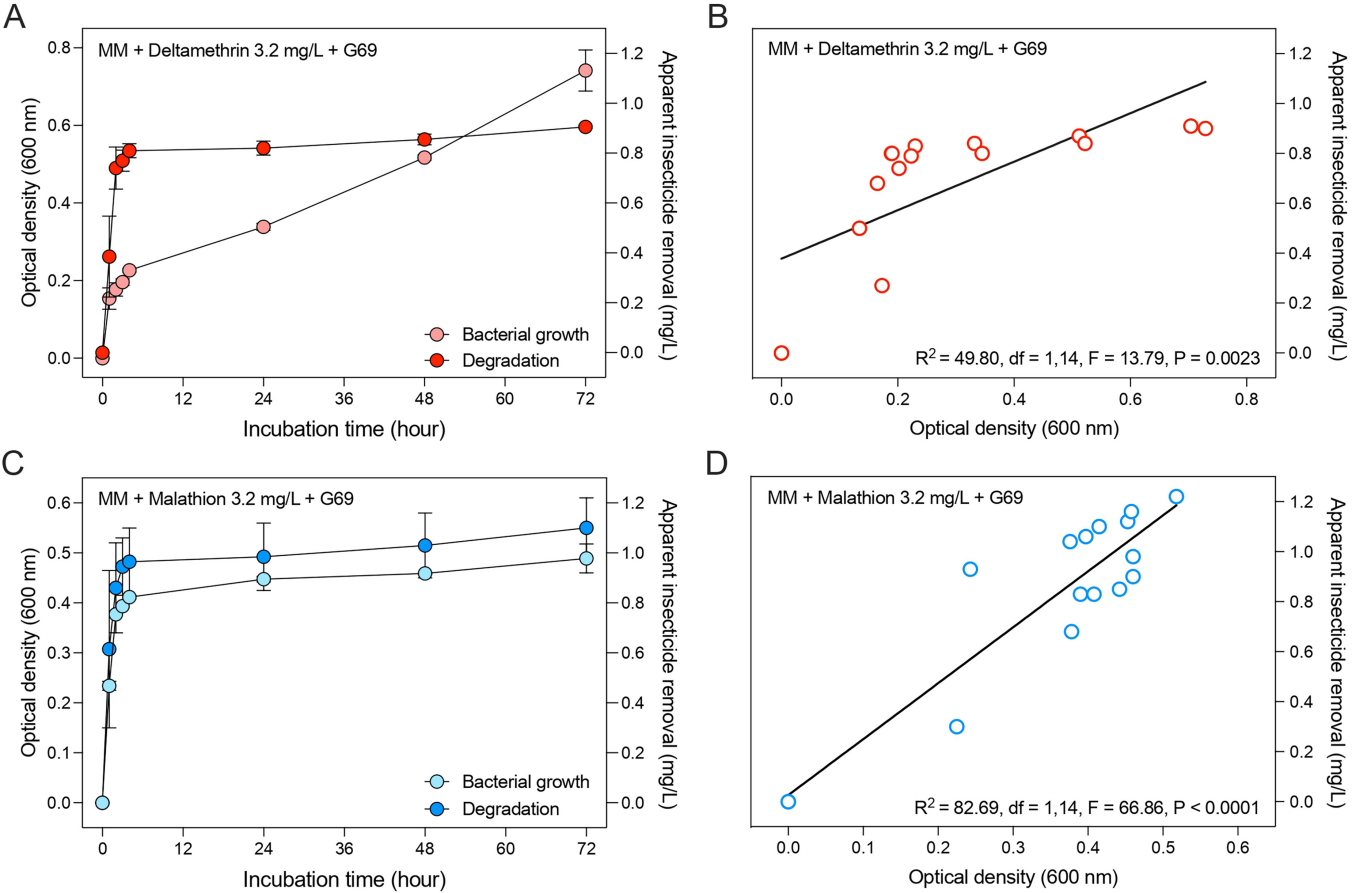
Apparent removal of parental insecticides in the presence of the actinobacterium *M. marinus* G96 strain. (**A**, **C**) Kinetics of bacterial growth, measured as optical density at 600 nm (OD_600_), and apparent insecticide removal (i.e., the combination of adsorption and degradation) of deltamethrin and malathion, respectively, by *M. marinus* G96, expressed in mg/L. Incubations are performed with 3.2 mg/L of the parent insecticides in MM liquid medium over 72 h. (**B**, **D**) Relationship between bacterial proliferation (OD_600_) and the apparent removal of parent deltamethrin and malathion (mg/L). OD_600_ values were normalized to the initial inoculum (time 0 = 0), whereas apparent insecticide removal was corrected for the analytical recovery, i.e., the concentration of the parent compound recovered in the corresponding abiotic controls.

Strain G96, identified as *Modestobacter marinus*, was the only isolate showing a consistent progressive increase in OD_600_ over time in the presence of 3.2 mg/L of this insecticide (Figure 1A, Supplementary Table 4), whereas the other strains displayed weak or inconsistent growth patterns (Supplementary Table 5). Such an increase in OD_600_ corresponded to a decrease in deltamethrin concentrations (Figure 1A; *R*^2^ = 0.4680, df = 1,14, *F* = 12.31, *P* = 0.0035; Figure 1B), with an apparent removal of 1.1 ± 0.17 mg/L (*n* = 2) after 72 h, representing 34.7% of the measured initial concentration detected at time 0 (3.17 mg/L, analytical recovery = 99.06%; apparent removal performance metrics in Supplementary Table 6). A similar growth-associated apparent removal pattern was observed for malathion. Among the tested strains, only G96 showed both a progressive increase in OD_600_ and a consistent decline in malathion concentrations (Figures 1C,D vs. Supplementary Tables 7, 8), with a significant positive correlation between the two (*R*^2^ = 0.8269, df = 1,14, *F* = 66.86, *P* < 0.0001). In this case, the overall malathion removal was lower, reaching 0.90 ± 0.01 mg/L at the end of the incubation, which corresponds to 30.4% of the measured initial concentration (2.98 mg/L; analytical recovery = 93.13%; other removal performance metrics in Supplementary Table 6).

Taken together, these results indicate that the decline in insecticide concentrations is not attributable solely to passive cell–insecticide interactions, such as adsorption to the bacterial surface or extracellular matrix. Static partitioning onto biomass alone would not produce a growth response in a minimal medium where the insecticide is the only organic carbon source provided (control result in Supplementary Figure 2C and OD_600_ in Supplementary Table 4). However, growth in minimal medium supplemented with insecticide consistently showed OD_600_ values below those achieved in rich medium (Supplementary Table 4) – which represents the strain’s optimal growth capacity under non-limiting nutrient conditions – suggesting that the utilization of the parent insecticides is energetically less favorable than growth on readily metabolizable substrates (e.g., sugars) for G96.

### Effect of insecticide concentration on the apparent removal by *Modestobacter marinus* G96

**FIGURE 2:**
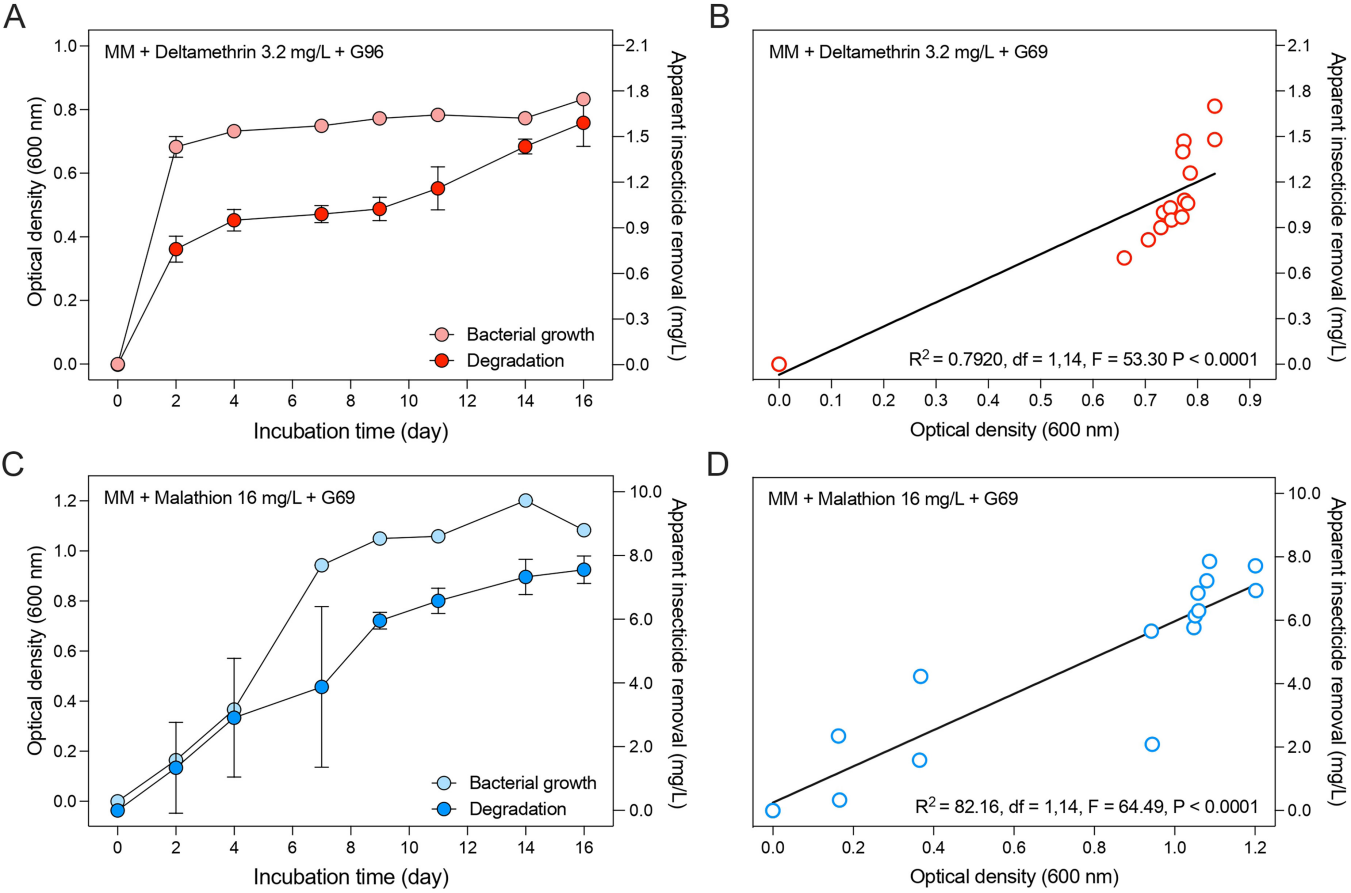
*Modestobacter marinus* G96 growth and apparent removal dynamics over 16 days of incubation. Bacterial growth, measured as optical density at 600 nm (OD_600_), and apparent pesticide removal (mg/L) were monitored during incubation of the G96 strain with (**A**, **B**) deltamethrin (3.2 mg/L) and (**C**, **D**) malathion (16 mg/L) over 16 days. OD_600_ values were normalized to the initial inoculum (time 0 = 0), whereas apparent insecticide removal was corrected for the analytical recovery, i.e., the concentration of the parent compound recovered in the corresponding abiotic controls. Exposure to higher concentrations of parental insecticides resulted in inhibition of bacterial growth; i.e., no clear patterns of consistent increases in OD_600_ and/or apparent insecticide removal were observed, as reported in Supplementary Figures S3 and S4.

To understand the long-term dynamics of apparent insecticide removal, we evaluated the performance of M. marinus G96 over a 16-day incubation period at three concentrations (3.2, 16, and 128 mg/L). This extended timeframe is critical, as pesticide bioavailability can decrease over time due to factors such as adsorption to biomass or container surfaces, crystallization, or chemical instability (Marín-Benito et al., 2017). For deltamethrin, no clear relationship between bacterial growth and insecticide apparent removal was observed at either 16 or 128 mg/L (Supplementary Figure 3). This decoupling suggests that the elevated deltamethrin concentrations likely limited bacterial metabolic activity and/or reduced insecticide bioavailability, preventing the growth-associated removal observed at lower concentrations. Such fluctuations in parent insecticide concentrations are indeed more consistent with passive, growth-independent processes, such as adsorption to the bacterial cell surface or extracellular matrix, rather than with active catabolism. In contrast, at the lowest concentration tested (3.2 mg/L), G96 exhibited a progressive increase in both OD_600_ and apparent malathion removal throughout the incubation period (Figures 2A,B). After 16 days, apparent removal reached 50.2% of the measured initial concentration (analytical recovery = 99.06%; removal performance metrics are reported in Supplementary Table 9). Yet, when G96 was cultivated in the presence of malathion, a significant pairing between bacterial growth (OD_600_) and apparent insecticide removal was maintained up to 16 mg/L (*R*^2^ = 0.8216, df = 1,14, *F* = 64.49, *P* < 0.0001; Figures 2C,D), indicating that bacterial proliferation remained closely associated with an increase in the apparent insecticide removal under these conditions. Over the 16-day incubation, the strain achieved a final reduction in parent insecticide of 7.55 ± 0.43 mg/L, corresponding to an apparent removal of 42.9% (analytical recovery = 85.63%; removal performance metrics in Supplementary Table 9). Strong inhibitory effects of malathion were observed at concentrations of 64 and 128 mg/L (see OD_600_ measurements in Supplementary Figure 4).

The observed differences in the utilization and/or tolerance of the two insecticides by G96 are consistent with well-documented structural and physicochemical distinctions between organophosphates and pyrethroids. Malathion is unusual among organophosphorus insecticides in possessing two additional carboxylic ester linkages alongside the phosphotriester bond common to all organophosphates (Supplementary Figure 1B) that are readily cleaved by carboxylesterases (EC 3.1.1.1), a broadly distributed bacterial enzyme class, yielding the less toxic malathion mono- and dicarboxylic acid derivatives as primary detoxification products (Singh and Walker, 2006; Ma et al., 2022). Deltamethrin, in contrast, is a Type II pyrethroid whose structure combines a halogenated (dibromovinyl) cyclopropane acid moiety with a bulky, cyano-substituted phenoxybenzyl alcohol group, conferring high hydrophobicity and very low aqueous solubility (Supplementary Figure 1A). This physicochemical profile favors strong partitioning onto surfaces rather than dissolution into the aqueous phase, thereby limiting its bioavailability, reducing access to hydrolytic enzymes, and slowing its biodegradation (Laskowski, 2002; Semple et al., 2003). Furthermore, pyrethroid biodegradation typically proceeds via initial ester-bond hydrolysis, releasing 3-phenoxybenzoic acid (3-PBA), a metabolite that is itself hydrophobic and persistent and can exert antibacterial effects that further constrain microbial growth and downstream degradation (Bhatt et al., 2019).

### Ecotoxicity of parent insecticides and their byproducts during incubation with strain G96

Toxicity assays using *A. salina* showed that the three treatments, i.e., supernatant of (i) bacterial culture in reach medium without insecticide, (ii) bacterial culture in MM with insecticide, and (iii) MM medium with insecticide without bacterium, significantly determine the rate of mortality over time (binomial GLM of treatment, likelihood ratio test deltamethrin: χ^2^ = 779.76, df = 2, *P* < 0.001 and likelihood ratio test malathion: χ^2^ = 762.37, df = 2, *P* < 0.001). Specifically, the G96 culture supernatant was non-toxic to *A. salina* (0% nauplii mortality), whereas the parent insecticides caused 100% nauplii mortality (Table 1). In contrast, the mortality of the byproducts presents in the culture supernatants varied over time, reflecting progressive detoxification (binomial GLM of incubation time, likelihood ratio test deltamethrin: χ^2^ = 142.83, *d* = 2, *P* < 0.001 and likelihood ratio test malathion: χ^2^ = 93.98, *d* = 2, *P* < 0.001). Pairwise comparisons using Fisher’s exact tests with Holm correction showed significant differences between all incubation times for the two insecticides (adjusted *P* < 0.05; Table 1). Immediately after inoculation (day 0), the insecticide-containing culture supernatants remained highly toxic, causing 100% mortality of *A. salina* when treated with the deltamethrin-treated supernatant and 77% mortality with the malathion-treated supernatant. Toxicity progressively declined during incubation with G96. After 7 days, mortality had decreased to moderate levels for both insecticides, and a further reduction was observed by day 14. At this time, the supernatant from the deltamethrin cultures caused only 26% mortality in *A. salina*, whereas the supernatant from the malathion cultures caused no detectable mortality under the tested conditions (Table 1). These results suggest that incubation with *M. marinus* G96 is associated with a marked reduction in toxicity over time, likely imputable to the formation of less toxic transformation products under the tested conditions. Also in this case, the difference in detoxification kinetics between deltamethrin and malathion may be explained by their chemical structures: deltamethrin degradation involves partial breakdown (i.e., incomplete mineralisation) of a halogenated aromatic structure, yielding stable and often more toxic intermediates/residues (Tang et al., 2020), whereas malathion degradation proceeds mainly via hydrolysis to small, hydrophilic, and less toxic compounds (Li et al., 2020).

**TABLE 1:** Toxicity of insecticide byproducts generated during incubation with *M. marinus* G96.

Sampling day	G96 alone	Deltamethrin		Malathion	
		No bacteria	G96	No bacteria	G96x
0	0 ± 0	100 ± 0	100 ± 0 (a)	100 ± 0	77 ± 7 (A)
7	0 ± 0	100 ± 0	69 ± 15 (b)	100 ± 0	32 ± 2 (B)
14	0 ± 0	100 ± 0	42 ± 15 (c)	100 ± 0	0 ± 0 (C)

Time-dependent survival of *A. salina* following exposure to deltamethrin or malathion and their respective byproducts generated during 14 days of incubation with *M. Marinus* G96 in MM medium supplemented with 3.2 mg/L of deltamethrin and 16 mg/L of malathion (i.e., the highest insecticide concentrations at which G96 maintained a significant association between bacterial growth and apparent insecticide removal). Survival percentages are reported as a mean and standard deviation from three independent replicates, each one including 30 individuals. Controls consist of supernatant from (i) G96 cultures in rich Ludemann medium without insecticides and (ii) MM medium supplemented with pesticides without bacteria, representing pesticide toxicity in the absence of removal over time. Within each treatment, different lowercase letters indicate significant differences among incubation times, as determined by pairwise Fisher’s exact tests with Holm-adjusted *P*-values (*P* < 0.05).

### Can the G96 strain remove parent malathion in soil slurry microcosms?

Given the results of toxicity tests, in which malathion degradation byproducts at 16 mg/L did not cause mortality in *A. salina* after 14 days of coincubation, we proceeded to evaluate the efficacy of *M. marinus* G96 in removing this insecticide from soil slurry. The strain was tested at two inoculum densities (5 × 10^8^ and 10^9^ CFU) in soil-slurry spiked with malathion at 64 and 128 mg/kg and incubated for 7, 14, and 21 days. Under both inoculum densities, G96 exhibited a high apparent removal capacity (Table 2; Supplementary Figure 5). At the 64 mg/kg treatment, apparent malathion removal reached 59.24 ± 0.14 mg/kg after 21 days with 5 × 10^8^ CFU and 57.26 ± 1.87 mg/kg after 14 days with 10^9^ CFU, corresponding to the removal of nearly all of the analytically recoverable insecticide detected in the soil-slurry abiotic control (recovery rate = 60.75 mg/kg, i.e., 94.92%). Similarly, at the 128 mg/kg treatment, apparent removal reached 100.18 ± 8.21 mg/kg after 14 days with 5 × 10^8^ CFU and 100.2 ± 2.21 mg/kg after 21 days with 10^9^ CFU. No significant differences in the measured removal were detected among the three incubation times (microcosms with 64 mg/L, Kruskal–Wallis’s test: *H* = 0.286, df = 2, *P* = 0.933; microcosms with 128 mg/L, Mann–Whitney U test: U = 2, *P* > 0.999) nor between bacterial density (Table 2).

**TABLE 2:** Quantification of apparent malathion removal (i.e., adsorption and degradation) in soil by *M. marinus* G96 at 7, 14, and 21 days.

Malathion added (mg/kg)	Recovery* (mg/kg, [%])	G96 inoculum (CFU)	Incubation time (days)	Apparent removal (mg/kg)	Normalized removal (mg/kg)
64	20.78 [94.92%]	5 × 10^8^	7	59.24 ± 0.015	55.99
		5 × 10^8^	14	59.72 ± 1.905	57.20
		5 × 10^8^	21	62.49 ± 0.141	59.24
		1 × 10^9^	14	60.50 ± 1.873	57.26
128	107.22 [83.76%]	5 × 10^8^	14	120.96 ± 2.220	100.18
		1 × 10^9^	7	118.97 ± 7.580	98.19
		1 × 10^9^	21	120.80 ± 2.220	100.02

Malathion was applied at concentrations of 64 and 128 mg/Kg, and the bacterial inoculum at concentrations of 5 × 10^8^ and 1 × 10^9^ cells. Microcosms without bacteria were included as abiotic controls to assess the recovery rate. Values are expressed as the average and standard deviation of two independent replicates per condition. *Recovery rate assessed in the abiotic controls with soil-slurry and melathion, but without the addition of bacterial inoculum.

### Putative malathion degradation pathway based on genome annotation and *in silico* analysis

**FIGURE 3:**
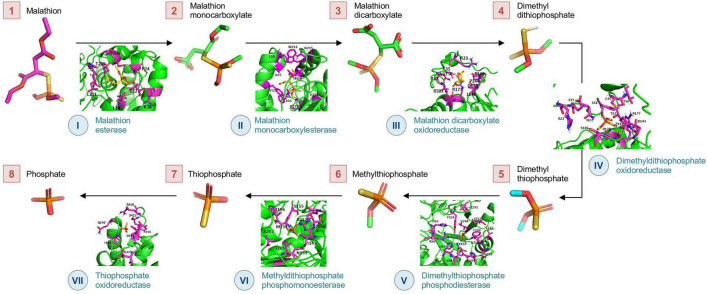
*In silico* analysis of the enzymes involved in the putative degradation pathway of malathion encoded by *M. marinus* G96. Each panel (I–VII) illustrates a catalytic step predicted by molecular docking, showing the progressive hydrolysis and oxidation of malathion into simpler, more polar intermediates. The enzyme structures are represented in green, whereas the substrates are shown in yellow. The catalytic residues involved in substrate binding and transformation are labeled in black, with their corresponding amino acid positions indicated. Molecular interactions and active-site residues were visualized and analyzed using PyMOL.

Using KO and EC numbers to assess the enzyme functions of all annotated genes, we reconstructed a putative malathion degradation pathway based on these functions in *M. marinus* G96. The molecular docking analysis supported the plausibility of enzyme-substrate interactions (Figure 3). The predicted pathway begins with the hydrolysis of malathion to a monocarboxylate by carboxylesterase (EC 3.1.1.1, KO: K01044), followed by hydrolysis of the monocarboxylate by the same enzyme to a dicarboxylate. After this step, the dicarboxylate is oxidized to dimethyl dithiophosphate by cytochrome P450 monooxygenases (EC 1.14.14.1, KO: K00490). The oxidation process is followed by a series of reactions that ultimately lead to dimethyl dithiophosphate, for which we identified two parallel pathways of organophosphate degradation. The first pathway involves hydrolysis through phosphodiesterase I (EC 3.1.4.1, KO: K01513), which produces phosphate and alcohol via glycerophosphocholine phosphodiesterase (EC 3.1.4.2, KO: K18695). The second pathway is a more direct dephosphorylation of dimethyl dithiophosphate to methanol and phosphate via alkaline phosphatase (EC 3.1.3.1, KO: K01077).

Overall, the genes encoding the key enzymes – cytochrome P450, carboxylesterase, and alkaline phosphatase – are distributed throughout the genome of *M. marinus*, suggesting that this bacterium likely degrades organophosphates through redundancy in the functional pathways described above. However, these findings remain predictive and hypothesis-generating.

## Discussion

Microbial transformation is a major component of the natural self-purification capacity of soils because microorganisms can convert xenobiotic compounds into less toxic intermediates and, in some cases, achieve their complete mineralisation to CO_2_, H_2_O and inorganic ions (Magan et al., 2010; Zhang and Zhang, 2022; Zhan, 2024). Unlike abiotic processes, microbial transformation is mediated by diverse enzymatic pathways that can respond dynamically to environmental conditions and contaminant availability (Karigar and Rao, 2011; Miglani et al., 2022; Yang et al., 2023; Zhou et al., 2023; Ren and Manefield, 2025). This adaptive metabolic capacity allows microbial communities to contribute to the long-term attenuation of contaminants while supporting the recovery of soil ecosystem functions in a far more sustainable and ecologically sound way than abiotic transformation alone (Lomeli-Ortega et al., 2024; Liu et al., 2025; Wu et al., 2025).

Despite the recognized role of microbial communities in pesticide transformation, most studies have focused on bacteria isolated from agricultural or contaminated soils (Cycoń and Piotrowska-Seget, 2016), whereas microorganisms inhabiting chronically nutrient-limited environments remain overlooked. This is particularly intriguing because persistent carbon and nutrient limitation is expected to favor microorganisms with broad metabolic plasticity and efficient resource-acquisition strategies, enabling them to exploit chemically diverse and often recalcitrant substrates when they become available (Props et al., 2019; Tong et al., 2024). Among these environments, lithic habitats such as desert rock surfaces and dust represent some of the most oligotrophic and environmentally stressful terrestrial ecosystems, where microorganisms experience chronic carbon limitation together with intense desiccation, solar radiation, and temperature fluctuations (Pointing and Belnap, 2012). These selective pressures have driven the evolution of highly resilient microbial communities with diverse physiological and metabolic capabilities (Leung et al., 2020). Within this ecological framework, the ability to transform anthropogenic xenobiotics should not be viewed as a direct evolutionary adaptation to synthetic pesticides, which are evolutionarily recent compounds, but rather as a potential consequence of the broad metabolic flexibility required for survival under persistent resource limitation (Marasco et al., 2023). Consequently, extremotolerant lithic actinobacteria represent a promising yet largely unexplored reservoir of metabolic traits that may be exploited to transform structurally diverse xenobiotics.

The present study was designed to test this ecological hypothesis by investigating whether actinobacteria isolated from desert rocks and dust (Saadouli et al., 2022) harbor previously unrecognized insecticide-removal capabilities. During the screening, we found that although nearly half of the isolates grew in minimal medium supplemented with one or both insecticides, only *Modestobacter marinus* G96 consistently achieved sustained bacterial growth with measurable apparent insecticide removal (i.e., a reduction in the parent insecticide concentration due to adsorption and/or biodegradation). This finding extends the functional range of the genus *Modestobacter* from passive stress-tolerant colonizers of barren substrates (Jiang et al., 2021) to potential participants in the detoxification of anthropogenic compounds. While the adaptation to rocky surfaces and desert dust is expected to favor microorganisms with broad metabolic flexibility and the capacity to exploit chemically diverse substrates (Belov et al., 2018; Mawang et al., 2021; Xie and Pathom-Aree, 2021), it does not necessarily imply specific evolution toward pesticide degradation, but it may increase the likelihood that these microorganisms possess enzymes capable of transforming such structurally complex xenobiotic compounds. Previous studies have indeed demonstrated pesticide transformation and/or degradation in several actinobacterial genera, including *Streptomyces*, *Rhodococcus*, *Microbacterium*, and *Micrococcus* (Guo et al., 2019; Lu P. et al., 2019; Dar et al., 2022; Jaiswal et al., 2025). For instance, *Micrococcus aloeverae* showed the highest malathion degradation at 500 μL/L within 15 days (Dar et al., 2022), while deltamethrin was degraded by *Streptomyces rimosus* up to 200 mg/L (Khajezadeh et al., 2020). However, reports describing microbial-mediated removal and/or transformation of both deltamethrin and malathion by a single microorganism remain uncommon and are largely restricted to members of the *Pseudomonadota* (Andleeb and Qazi, 2014) and *Bacillota* (Wageed et al., 2021). The identification of a member of the *Actinomycetota* capable of doing it therefore expands the taxonomic diversity of microorganisms potentially involved in the attenuation of such insecticides.

The limited number and taxonomic diversity of bacterial strains reported to transform both malathion and deltamethrin may, at least in part, reflect the fundamentally different physicochemical properties and degradation requirements of these insecticides. Malathion is an organophosphate that is relatively susceptible to hydrolytic cleavage (Singh and Walker, 2006; Jensen and Whatling, 2010), whereas deltamethrin is a highly hydrophobic pyrethroid possessing a highly hydrophobic halogenated aromatic structure that strongly adsorbs to organic matter and biological surfaces, thereby reducing its aqueous bioavailability (Marín-Benito et al., 2017). Consistent with these properties, G96 exhibited a stronger growth-associated relationship with malathion removal than with deltamethrin at elevated concentrations, suggesting that reduced bioavailability and/or inhibitory effects of the latter may have limited G96 performance. These observations are consistent with the known environmental behavior of pyrethroids (Laskowski, 2002; Cycoń and Piotrowska-Seget, 2016), although direct measurements of adsorption, metabolite formation, and mineralization will be required to confirm the proposed mechanisms.

Alongside the growth-associated apparent removal of the parent insecticides, the marked reduction in toxicity observed during incubation with *M. marinus* G96 provides an independent line of evidence consistent with microbial transformation of the tested insecticides. In particular, the complete loss of acute toxicity of the malathion-treated culture supernatants toward *A. salina* after 14 days indicates that the insecticide-containing medium was substantially detoxified during incubation with G96. Although the chemical identity of the transformation products was not determined, the marked reduction in toxicity, together with the concomitant decrease in the parent insecticide concentration, is consistent with microbial biotransformation rather than simple physical sequestration. This process may involve hydrolytic reactions that generate less toxic, more readily metabolized intermediates that can subsequently enter central metabolic pathways and biogeochemical cycles (Chen et al., 2011; Ma et al., 2022), although confirmation of these pathways will require targeted metabolite identification and mineralization assays. Our genome annotation and molecular docking analyses indeed identified several candidate enzymes that may participate in at least the transformation of malathion (Figure 3), thereby providing a plausible mechanistic framework for the observed phenotype. Such enzymes lead to the formation of more hydrophilic derivatives, including phosphate and fumarate, which readily integrate into natural microbial and nutrient turnover (Kumar et al., 2019; Dar and Kaushik, 2023). However, these analyses are predictive and should be regarded as hypothesis-generating rather than direct evidence of enzymatic activity. To move beyond these theoretical interpretations, experimental confirmation through transcriptomic analyses, enzyme purification, gene knockout approaches, or heterologous expression will be required to establish the specific catalytic mechanisms encoded by G96.

Importantly, the present study was designed as a proof-of-concept to identify previously unexplored actinobacteria capable of removing insecticides under nutrient-limited conditions rather than to fully resolve the mechanisms responsible for insecticide removal. Although bacterial growth, reduction of the parent compounds measured by HPLC, detoxification in *A. salina*, and successful removal in soil microcosms together provide strong evidence that G96 actively contributes to insecticide transformation, the current experimental design does not allow unequivocal discrimination between biodegradation, biosorption, or other removal processes. Future investigations should therefore combine abiotic sorption controls, metabolomic profiling, enzymatic characterization, and mineralization assays to quantify the relative contributions of adsorption and biodegradation, identify transient transformation products, and elucidate the complete metabolic pathways responsible for insecticide transformation by G96. Such studies will also clarify the influence of physicochemical properties, including hydrophobicity and bioavailability, on the efficiency of growth-associated pesticide removal.

## Conclusion

This study identifies *M. marinus* G96 as a previously unrecognized actinobacterium capable of growth-associated removal of two chemically distinct insecticides, deltamethrin and malathion, under nutrient-limited conditions. Apparent insecticide removal was accompanied by a marked reduction in toxicity and was sustained in soil-slurry microcosms, supporting this strain’s ability to actively attenuate insecticides under environmentally relevant conditions. Although the present data do not permit unequivocal discrimination between biodegradation, biosorption, and other removal processes, they provide the first proof-of-concept that extremotolerant actinobacteria inhabiting oligotrophic lithic environments represent a previously underexplored reservoir of metabolic functions relevant to pesticide removal. In fact, the discovery of this phenotype extends the recognized functional diversity of the genus *Modestobacter* beyond its established role as a stress-tolerant colonizer of nutrient-poor mineral substrates. More broadly, our findings support the hypothesis that adaptation to chronically oligotrophic and physically harsh environments favors metabolic flexibility that may incidentally enable the transformation of anthropogenic xenobiotics. This ecological framework suggests that lithic microbial communities constitute a valuable source of novel catabolic traits that remain largely unexplored.

Together, these findings establish a foundation for future mechanistic investigations aimed at resolving the enzymatic pathways, regulatory networks, metabolite formation, and the relative contributions of biodegradation and adsorption to insecticide removal. Such knowledge will be essential for evaluating the broader substrate range of G96 and related extremotolerant actinobacteria and for determining their suitability as biological tools for the sustainable remediation of pesticide-contaminated soils. This opportunity is particularly relevant for dryland agroecosystems, where environmental stress and limited water availability can constrain conventional remediation strategies, and where harnessing naturally adapted microorganisms may provide an effective, environmentally compatible alternative to restoring soil quality and ecosystem function.

## Data Availability

The datasets presented in this study can be found in online repositories. The names of the repository/repositories and accession number(s) can be found in the article/Supplementary material.

## Footnotes

1
https://www.kegg.jp/kegg/pathway.html

2
https://eawag-bbd.ethz.ch/mal/mal_map.html
