# Supplementary material for: Novel insecticide-removal activity against deltamethrin and malathion in a lithic-derived Modestobacter strain
Source: Front Microbiol. 2026 Jul 29;17:1871101. doi: 10.3389/fmicb.2026.1871101 (PMC13462060; doi:10.3389/fmicb.2026.1871101)
Supplement: Supplementary file 1 [file Table_2.docx]

Supplementary Material of

**Novel insecticide-removal activity against deltamethrin and malathion in a lithic-derived *Modestobacter* strain**

Tasnim HAJRI^1^, Ramona MARASCO^2^, Ilhem SAADOULI^3^, Khouloud HAMMAMI^1^, Hanen CHERIF^1^, Wafa BABAY^3^, Soumaya KOUIDHI^1^, Hadda-Imene OUZARI^3^, Meriem MSAAD^4^, George TSIAMIS^5^, Ameur CHERIF^1^, and Amor MOSBAH^1^*

^1^Laboratory of Biotechnology and Bio-Geo Resources Valorisation (LR11ES31), Higher Institute of Biotechnology of Sidi Thabet (ISBST), University of Manouba, 2010, Tunis, Tunisia

^2^Biological and Environmental Sciences and Engineering Division, King Abdullah University of Science and Technology, Thuwal 23955-6900, Saudi Arabia

^3^Laboratory of Microorganisms and Active Biomolecules (LR03ES03), Faculty of Sciences of Tunis (FST), University Tunis El Manar,2092, Tunis, Tunisia

^4^Laboratory of Biotechnology and Nuclear Technologies (LR16CNSTN01), National Centre for Nuclear Science and Technology, Technological Pole – 2020 Sidi Thabet BP 72, Tunis, Tunisia

^5^Laboratory of Systems Microbiology and Applied Genomics, Department of Sustainable Agriculture, University of Patras, Agrinio, Greece

*Corresponding author: Amor MOSBAH: [amor.mosbah@isbst.uma.tn](mailto:amor.mosbah@isbst.uma.tn)

**Supplementary Figures**

**Supplementary Figure S1.** Molecular structure of (**A**) deltamethrin and (**B**) malathion. Image reproduced from PubChem.

(**A**)

(**B**)

**Supplementary Figure S2.** Representative images of the primary screening assay used to evaluate the ability of 70 actinobacterial isolates to grow on minimal medium (MM) supplemented with insecticides as the sole carbon source. Plates contained MM supplemented with (**A**) deltamethrin or (**B**) malathion, while (**C**) MM without insecticides served as the negative control. Growth was considered positive when a continuous bacterial colony developed along the entire inoculation streak, indicating the strain’s ability to utilise the insecticide under nutrient-limited conditions. Representative strains exhibiting positive growth are labelled, illustrating the variability in growth responses among the screened isolates.

(**A**) MM + deltamethrin

(**B**) MM + malathion

(**C**) MM without insecticide

**Supplementary Figure S3.** Growth of *Modestobacter marinus* G96 and degradation of deltamethrin during 16 days of incubation. Bacterial growth (OD_600_) and deltamethrin concentration are shown for cultures initially supplemented with (**A, B**) 16 mg/L or (**C, D**) 128 mg/L of deltamethrin. OD_600_ values were normalised to the initial inoculum, giving a starting value of zero. Deltamethrin concentrations were corrected for analytical recovery using the measured concentration at T0 as the baseline for all subsequent calculations. Recovery rates of the abiotic controls were 90.6% and 33.8% of the initial concentrations for 16 mg/L and 128 mg/L treatments, respectively.

**Supplementary Figure S4.** Bacterial growth (OD_600_) of *Modestobacter marinus* G96 during 16 days of incubation on (**A**) 64 mg/L and (**B**) 128 mg/L of malathion. OD_600_ values were normalised to the initial inoculum, giving a starting value of zero.

**Supplementary Figure S5.** HPLC chromatographic analysis of crude extract obtained after malathion extraction from soil.

**Supplementary Tables**

**Supplementary Table S1.** Analytical validation parameters for the HPLC quantification of malathion and deltamethrin. Linear regression analysis was performed over a concentration range of 1-5 mg/L. R^2^ represents the coefficient of determination; a and b are the slope and intercept of the regression equation (y = axe + b), respectively. The limit of Detection (LOD) and Limit of Quantification (LOQ) were calculated using the formulas 3.3 σ/S and 10 σ/S, where σ is the standard deviation of the y-intercept, and S is the slope. Sandell’s sensitivity (SS) is expressed in g/cm/0.001/Abs.unit.

| **Parameters** | **Malathion** | **Deltamethrin** |
| --- | --- | --- |
| **Calibration range (mg/L)** | 1-5 | 1-5 |
| **Regression equation (Y=ax+b)** | Y= 306707X+3217866.2 | Y= 2598475.4X+8545088.4 |
| **R^2^ values** | 0.9693 | 0.9695 |
| **Slope (a)** | 306707 | 2598475.4 |
| **Intercept (b)** | 3217866.2 | 8545088.4 |
| **Sandell’s sensitivity (SS)*** | 3.26×10^−9^ | 3.85×10−10 |
| **Limit of Detection (LOD) (mg/L)** | 1.12 | 1.12 |
| **Limit of quantification (LOQ) (mg/L)** | 3.41 | 3.39 |

*Sandell’s sensitivity expressed as g/cm²/0.001/Abs. unit.

**Supplementary Table S2.** Physicochemical characteristics of the soil collected from Sidi Thabet and used for microcosm experiments. The analyses were conducted at the Mediterranean Centre for Environmental and Industrial Analyses following standard protocols.

| **Soil property (unit)** | **Value** |
| --- | --- |
| pH | 8.7 |
| Temperature (°C) | 21.2 |
| Conductivity at 25°C (μS/cm) | 177 |
| Chlorides (mg/Kg DM) | 31 |
| Organic Matter (g/Kg DM) | 85.5 |
| Dry Matter (%) | 96.5 |
| Magnesium (g/Kg DM) | 6.07 |
| Calcium (g/Kg DM) | 151.4 |
| Sodium (g/Kg DM) | 1.09 |
| Potassium (g/Kg DM) | 6.77 |
| Sand (%) | 31.2 |
| Clay (%) | 43.8 |
| Silt (%) | 22.7 |
| Orthophosphate (mg PO4/Kg DM) | 1.92 |
| Total Nitrogen (mg N/Kg DM) | 0.8 |
| Total Organic Carbon (g/Kg DM) | 50.3 |
| Sulfate (mg/Kg DM) | < 2.0 |

DM: Dry Matter

**Supplementary Table S3.** Results of the growth-screening of 70 actinobacterial strains cultivated in minimal medium in the presence of the two insecticides, i.e., deltamethrin (D) and malathion (M). Results of growth on minimal medium alone (C) are also reported as a control to assess the utilisation of medium components and/or agar as carbon sources.

| **Strain** | **Taxonomy** | **D** | **M** | **C** | **Strain** | **Taxonomy** | **D** | **M** | **C** |
| --- | --- | --- | --- | --- | --- | --- | --- | --- | --- |
| G1 | *Streptomyces sampsonii* | + | + | + | G29 | *Arthrobacterglobiformis* | - | + | + |
| G2 | *Streptomyces flavoviridis* | + | + | + | G30 | *Streptomyces moderatus* | + | + | - |
| K2 | *Kocuria* sp. | - | - | - | K30 | *Kocuria* sp. | + | + | - |
| G3 | *Streptomyces* sp. | + | - | + | G31 | *Cellulomonascellasea* | - | - | + |
| K3 | *Planomicrobiumokeanokoites* | - | - | - | G32 | *Cellulomonas* sp. | - | - | + |
| G5 | *Streptomyces* sp. | - | + | + | K32 | *Kocuria polaris* | - | - | + |
| P5 | *Glutamicibacter creatinolyticus* | - | + | - | G33 | *Enterobacter* sp*.* | - | - | - |
| P6 | *Cronobacter sakazakii* | - | + | - | K33 | *Kocuria* sp. | - | - | + |
| G7 | *Streptomyces mutabilis* | + | - | + | G34 | *Kocuria turfanensis* | - | - | + |
| G8 | *Streptomyces hydrogenans* | - | - | + | G36 | *Auraticoccus* sp. | - | - | - |
| K8 | *Kocuria* sp. | - | - | - | G37 | *Arthrobacter subterraneus* | - | - | + |
| G9 | *Streptomyces* sp. | + | + | - | G39 | *Arthrobacter globiformis* | - | - | + |
| G10 | *Cellulomonascellasea* | - | - | - | G40 | *Streptomyces calvus* | + | - | + |
| G11 | *Streptomyces roietensis* | - | - | - | G42 | *Streptomyces* sp. | + | - | - |
| G12 | *Arthrobacter* sp. | - | - | + | G44 | *Streptomyces* sp. | - | + | - |
| G13 | *Kocuria* sp. | + | + | - | G47 | *Streptomyces* sp. | - | + | + |
| G13.B | *Brevibacteriumfrigoritolerans* | + | - | + | G48 | *Streptomyces fradiae* | + | + | - |
| G13a2 | *Corynebacterium stationis* | - | - | - | G55 | *Streptomyces* sp. | - | + | - |
| G14 | *Rhizobium sp.* | - | + | - | G59 | *Arthrobacter* sp. | - | - | + |
| G16 | *Agrococcus jenensis* | + | - | + | G60 | *Kribbella catacumbae* | + | + | - |
| K16 | *Kocuria* sp. | - | - | - | G61 | *Auraticoccus monumenti* | - | - | - |
| 16.D | *Streptomyces luteus* | + | + | - | G62 | *Arthrobacter* sp. | - | - | + |
| G17 | *Cellulosimicrobiumfunkei* | - | - | - | G63 | *Arthrobacter* sp. | - | - | + |
| G17.14 | *Pseudarthrobacter phenanthrenivorans* | - | - | - | G65 | *Agrococcus* sp. | - | - | - |
| G17.15 | *Kocuria rhizophila* | + | + | - | G69 | *Streptomyces* sp. | + | + | - |
| G17.I | *Acinetobacter johnsonii* | - | - | - | G70 | *Auraticoccus monumenti* | - | - | + |
| G21 | *Rhizobium* sp. | - | - | - | G71 | *Arthrobacter tumbae* | - | - | - |
| G22 | *Arthrobacter* sp. | - | - | + | G76 | *Streptomyces* sp. | - | - | - |
| G23 | *Streptomyces* sp. | + | - | + | G79 | *Streptomyces* sp. | + | + | - |
| G25 | *Streptomyces* sp. | - | - | - | G81 | *Streptomyces venezuelae* | + | - | + |
| G26 | *Nocardia* sp. | - | + | + | G89 | *Jiangella mangrovi* | + | + | - |
| G27 | *Methylobacterium* sp. | - | - | + | G90 | *Nocardia* sp*.* | - | - | + |
| K27 | *Kocuria rosea* | - | - | + | G91 | *Auraticoccus* sp*.* | - | - | - |
| G28 | *Staphylococcus succinus* | - | - | - | G93 | *Streptomyces* sp. | + | - | + |
| K28 | *Micrococcus lylae* | - | - | + | G96 | *Modestobacter marinus* | + | + | - |

(+): growth, (-): no growth

**Supplementary Table S4.** Optical density (OD_600_) of *Modestobacter marinus* G96 during 72 h of incubation in different growth media. Cultures were incubated in Luedemann rich medium, minimal medium (MM) supplemented with 3.2 mg/L malathion, MM supplemented with 3.2 mg/L deltamethrin, or MM without insecticide (control). OD_600_ values are reported for two independent biological replicates and were normalised to the initial inoculum, giving a starting value of zero.

| **Time (h)** | **Luedemann**  **(rich-medium)** | | **MM with**  **malathion (3.2 mg/L)** | | **MM with**  **delthametrin (3.2 mg/L)** | | **Minimal medium (control)** | |
| --- | --- | --- | --- | --- | --- | --- | --- | --- |
|  | **Rep 1** | **Rep 2** | **Rep 1** | **Rep 2** | **Rep 1** | **Rep 2** | **Rep 1** | **Rep 2** |
| 0 | 0 | 0 | 0 | 0 | 0 | 0 | 0 | 0 |
| 1 | 0.463 | 0.462 | 0.173 | 0.034 | 0.243 | 0.225 | 0.008 | 0.007 |
| 2 | 0.58 | 0.585 | 0.189 | 0.065 | 0.376 | 0.378 | 0.016 | 0.017 |
| 3 | 0.619 | 0.601 | 0.190 | 0.102 | 0.397 | 0.39 | 0.028 | 0.028 |
| 4 | 0.666 | 0.642 | 0.230 | 0.123 | 0.415 | 0.408 | 0.026 | 0.038 |
| 24 | 0.815 | 0.739 | 0.332 | 0.245 | 0.453 | 0.442 | 0.021 | 0.022 |
| 48 | 1.686 | 1.452 | 0.512 | 0.422 | 0.458 | 0.460 | 0.030 | 0.041 |
| 72 | 1.292 | 1.264 | 0.704 | 0.729 | 0.518 | 0.475 | 0.038 | 0.042 |

**Supplementary Table S5.** Measurements of optical density at 600 nm over 72 hours of 13 actinobacterial strains incubated in minimal medium added with 3.2 mg/L of deltamethrin. Values are reported for two independent incubations. OD_600_ values were normalised to the initial inoculum, giving a starting value of zero. Strains showing a consistent increase in OD_600_ over the incubation period are highlighted in bold, indicating those selected as positive candidates for subsequent removal/degradation assays by HPLC.

| **Strain** | **Time (hours)** | | | | | | | |
| --- | --- | --- | --- | --- | --- | --- | --- | --- |
|  | **0** | **1** | **2** | **3** | **4** | **24** | **48** | **72** |
| G1 *Streptomyces sampsonii* | 0 | 0 | 0 | 0.008 | 0 | 0 | 0.064 | 0.176 |
|  | 0 | 0.178 | 0 | 0.01 | 0.105 | 0.297 | 0.262 | 0.257 |
| G2 *Streptomyces flavoviridis* | 0 | 0 | 0 | 0 | 0 | 0.562 | -0.344 | 0.952 |
|  | 0 | 0.049 | 0.046 | 0 | 0 | 0.135 | 0.991 | 1.048 |
| G3 *Streptomyces* sp. | 0 | 0 | 0 | 0 | 0 | 0 | 0 | 0 |
|  | 0 | 0.053 | 0.077 | 0.006 | 0 | 0 | 0 | 0 |
| G7 *Streptomyces mutabilis* | 0 | 0 | 0 | 0 | 0 | 0 | 0 | 0.138 |
|  | 0 | 0.029 | 0.729 | 0 | 0 | 0 | 0 | 0.161 |
| G9 *Streptomyces* sp. | 0 | 0 | 0 | 0 | 0 | 0 | 0 | 0 |
|  | 0 | 0 | 0 | 0 | 0 | 0 | 0.124 | 0.268 |
| G23 *Streptomyces* sp. | 0 | 0 | 0 | 0 | 0 | 0 | 0 | 0.093 |
|  | 0 | 0 | 0 | 0 | 0 | 0 | 0.021 | 0.165 |
| G40 *Streptomyces calvus* | 0 | 0 | 0 | 0 | 0 | 0 | 0 | 0 |
|  | 0 | 0 | 0 | 0 | 0 | 0 | 0.108 | 0 |
| G48 *Streptomyces fradiae* | 0 | 0 | 0 | 0 | 0 | 0 | 0 | 0 |
|  | 0 | 0 | 0 | 0 | 0 | 0 | 0 | 0 |
| G60 *Kribbella catacumbae* | 0 | 0 | 0 | 0 | 0 | 0.015 | 0 | 0.225 |
|  | 0 | 0 | 0.365 | 0.498 | 0.245 | 0 | 0.122 | 0.231 |
| G81 *Streptomyces venezuelae* | 0 | 0 | 0 | 0 | 0 | 0 | 0 | 0 |
|  | 0 | 0 | 0 | 0 | 0 | 0 | 0.024 | 0 |
| G89 *Jiangella mangrovi* | 0 | 0 | 0 | 0 | 0 | 0.021 | 0 | 0 |
|  | 0 | 0 | 0.043 | 0 | 0 | 0 | 0.141 | 0.061 |
| G93 *Streptomyces* sp. | 0 | 0.028 | 0 | 0.031 | 0.058 | 0 | 0 | 0 |
|  | 0 | 0 | 0.113 | 0.009 | 0.058 | 0 | 0 | 0 |
| **G96 *Modestobacter marinus*** | **0** | **0.243** | **0.376** | **0.397** | **0.415** | **0.453** | **0.458** | **0.518** |
|  | **0** | **0.225** | **0.378** | **0.39** | **0.408** | **0.442** | **0.46** | **0.460** |

**Supplementary Table S6.** Summary of apparent removal kinetics of deltamethrin and malathion by *Modestobacter marinus* G96 over 72 hours of incubation. Kinetic parameters were estimated using a one-phase asymptotic removal model. Values should be interpreted as apparent removal kinetics because the present dataset does not distinguish biodegradation from biosorption or other non-biological removal processes.

| **Parameter** | **Deltamethrin** | **Malathion** |
| --- | --- | --- |
| Initial concentration | 3.2 mg L⁻¹ | 3.2 mg L⁻¹ |
| Measured concentration t_0_ | 3.17 mg L⁻¹ | 2.98 mg L⁻¹ |
| Analytical recovery | 99.06% | 93.13% |
| Net removal at 72 h | 1.10 ± 0.17 mg L⁻¹ | 0.905 ± 0.007 mg L⁻¹ |
| Net removal (%) | 34.7% | 30.4% |
| Removal at 2 h | 0.86 mg L⁻¹ / 27.1% | 0.74 mg L⁻¹ / 24.8% |
| Removal at 4 h | 0.965 mg L⁻¹ / 30.4% | 0.81 mg L⁻¹ / 27.2% |
| Initial removal rate (0–4 h) | 0.241 mg L⁻¹ h⁻¹ | 0.203 mg L⁻¹ h⁻¹ |
| Apparent rate constant (*k*) | 0.719 ± 0.238 h⁻¹ | 0.738 ± 0.091 h⁻¹ |
| Apparent half-time (t₁/₂) | 0.96 h | 0.94 h |
| Time to 90% of plateau (t₉₀) | 3.20 h | 3.12 h |

**Supplementary Table S7.** Optical density (OD_600_) measurements of eight actinobacterial strains incubated in minimal medium supplemented with 3.2 mg/L malathion over 72 hours. Values are reported for two independent biological replicates. OD_600_ values were normalised to the initial inoculum, giving a starting value of zero. Strains showing a consistent increase in OD_600_ over the incubation period are highlighted in bold, indicating those selected as positive candidates for subsequent removal/degradation assays by HPLC (see **Supplementary Table S7**).

| **Strain** | **Time (hours)** | | | | | | | |
| --- | --- | --- | --- | --- | --- | --- | --- | --- |
|  | **0** | **1** | **2** | **3** | **4** | **24** | **48** | **72** |
| G1 *Streptomyces sampsonii* | 0 | 0.001 | 0.005 | 0.003 | 0.002 | 0.003 | 0.001 | 0.001 |
|  | 0 | 0.001 | 0.001 | 0.002 | 0.001 | 0 | 0.003 | 0.002 |
| G2 *Streptomyces flavoviridis* | 0 | 0.296 | 0.384 | 0.287 | 0.235 | 0.557 | 0.526 | 0.382 |
|  | 0 | 0.300 | 0.340 | 0.429 | 0.374 | 0.455 | 0.166 | 0.145 |
| **P5 *Glutamicibacter creatinolyticus*** | **0** | **0.596** | **0.619** | **0.932** | **0.971** | **1.044** | **0.951** | **0.933** |
|  | **0** | **0.596** | **0.619** | **0.932** | **0.991** | **1.041** | **1.002** | **0.928** |
| **P6 *Cronobacter sakazakii*** | **0** | **0.053** | **0.126** | **0.236** | **0.362** | **0.49** | **0.6** | **0.58** |
|  | **0** | **0.064** | **0.114** | **0.221** | **0.357** | **0.471** | **0.599** | **0.579** |
| **G48 *Streptomyces fradiae*** | **0** | **0.082** | **0.162** | **0.203** | **0.249** | **0.543** | **0.719** | **0.573** |
|  | **0** | **0.077** | **0.165** | **0.198** | **0.229** | **0.453** | **0.648** | **0.543** |
| G60 *Kribbella catacumbae* | 0 | 0.057 | 0.153 | 0.111 | 0.126 | 0.322 | 0.495 | 0.326 |
|  | 0 | 0.255 | 0.163 | 0.320 | 0.311 | 0.347 | 0.351 | 0.328 |
| G89 *Jiangella mangrovi* | 0 | 0.268 | 0.075 | 0.335 | 0.088 | 0.099 | 0.213 | 0.144 |
|  | 0 | 0.068 | 0.152 | 0.054 | 0.177 | 0.221 | 0.05 | 0.124 |
| **G96 *Modestobacter marinus*** | **0** | **0.173** | **0.189** | **0.19** | **0.23** | **0.332** | **0.512** | **0.704** |
|  | **0** | **0.034** | **0.065** | **0.102** | **0.123** | **0.245** | **0.422** | **0.729** |

**Supplementary Table S8.** Removal of malathion during incubation with actinobacterial strains exhibiting a progressive increase in OD_600_ (see **Supplementary Tables** **S6**). The concentration of malathion in the medium was determined by HPLC with peak-area quantification. Insecticide removal, encompassing both adsorption and biodegradation, is expressed as mg/L. Concentration values were normalised using the insecticide concentration measured at time 0 as the baseline. The recovery rate in the presence of 3.2 mg/L malathion was 93.1%.

| **Time (h)** | **Strain P5** | |  | **Strain P6** | |  | **Strain G48** | |
| --- | --- | --- | --- | --- | --- | --- | --- | --- |
|  | **Rep1** | **Rep2** |  | **Rep1** | **Rep2** |  | **Rep1** | **Rep2** |
| 0 | 0 | 0 |  | 0 | 0 |  | 0 | 0 |
| 1 | 0.85 | 0.58 |  | 0.1 | 0.22 |  | 0.04 | 0.03 |
| 2 | 0.33 | 0.18 |  | 0.8 | 0.45 |  | 0.33 | 0.24 |
| 3 | 0.5 | 0 |  | 0.24 | 0.48 |  | 0.26 | 0.23 |
| 4 | 0.26 | 0.15 |  | 1.17 | 1.3 |  | 0.39 | 0.21 |
| 24 | 0.58 | 0.41 |  | 0.95 | 0.55 |  | 0.32 | 0.3 |
| 48 | 0.61 | 0.12 |  | 1.2 | 1.79 |  | 0.39 | 0.18 |
| 72 | 0.34 | 0.17 |  | 0.11 | 0.30 |  | 0.21 | 0.63 |

**Supplementary Table S9.** Summary of apparent kinetic parameters describing the net removal of deltamethrin (3.2 mg/L) and malathion (16 mg/L) by *Modestobacter marinus* G96 estimated over 16 days of incubation. Removal calculations were based on the experimentally measured concentrations in abiotic controls at time 0 rather than the nominal concentrations added, i.e., 13.7 mg/L [85.6%] and 3.17 mg/L [99.06%], respectively. Kinetic parameters were estimated by fitting the data to a one-phase asymptotic removal model. These parameters should not be interpreted as biodegradation kinetics alone, because biosorption and biodegradation were not quantified independently.

| **Parameter** | **Deltamethrin** | **Malathion** |
| --- | --- | --- |
| Initial concentration | 3.2 mg L⁻¹ | 16 mg L⁻¹ |
| Measured concentration t_0_ | **3.17 mg L⁻¹** | **13.7 mg L⁻¹** |
| Analytical recovery | **99.06%** | **85.6%** |
| Net removal at 16 d | **1.59 ± 0.16 mg L⁻¹** | **7.56 ± 0.43 mg L⁻¹** |
| Net removal (%) | **50.2%** | **55.2%** |
| Removal at 2 d | **0.95 ± 0.07 mg L⁻¹ (30.0%)** | **2.91 ± 1.87 mg L⁻¹ (21.2%)** |
| Removal at 4 d | **1.03 ± 0.08 mg L⁻¹ (32.3%)** | **5.96 ± 0.26 mg L⁻¹ (43.5%)** |
| Initial removal rate (0–4 d) | **0.256 mg L⁻¹ d⁻¹** | **1.49 mg L⁻¹ d⁻¹** |
| Apparent rate constant (k) | **0.499 d⁻¹** | **≈ 0.67 d⁻¹** |
| Apparent half-time (t₁/₂) | **1.39 d** | **≈ 1.03 d** |
| Time to 90% of plateau (t₉₀) | **4.61 d** | **≈ 3.4 d** |
